# Laboratory medicine resilience during coronavirus disease 2019 (COVID-19) pandemic

**DOI:** 10.1515/almed-2020-0035

**Published:** 2020-04-21

**Authors:** Giuseppe Lippi, Mario Plebani

**Affiliations:** Section of Clinical Biochemistry University Hospital of Verona Piazzale L.A. Scuro, 10 37134 Verona, Italy; Department of Laboratory Medicine, University Hospital of Padova, Padova, Italy

*In vitro* diagnostic testing has been a virtually innocent target of heavy, reiterated, often irrational cost-containment polices during the past decades, which have contributed to diminishing the availability of technical and human resources all around the world [1]. Although working close to the “minimal level of survival” has hence become commonplace in the vast majority of laboratory services [2], the coronavirus disease 2019 (COVID-19) pandemic has triggered an unexpected and unprecedented universal crisis, which has rapidly overwhelmed the responsive capacity of the entire system of health care, thus also including laboratory diagnostics [3]. The last report of the World Health Organization emphasizes that COVID-19 has already affected more than 1 million individuals around the world, causing over 50,000 deaths [4]. The epidemiological figures in some Countries like Spain and Italy are especially alarming, whereby the number of affected people has rapidly grown over 100,000, with mortality rates as high as 8–11% [4], thus closely reflecting that of the previous severe acute respiratory syndrome (SARS) outbreak, caused by an analogous coronavirus [5]. Even more importantly, nearly 20% of all COVID-19 cases require hospitalization for either sub-intensive or intensive care, thus posing an additional burden to clinical laboratories, which are forced to produce a large volume of critical (i. e., urgent) tests results, with the shortest possible turnaround time [6].

In this “perfect storm” scenario, laboratory medicine is once more demonstrating its inherent and well-known resilience, whereby laboratory professionals are uninterruptedly providing vital test results for diagnosing, prognosticating and managing patients with COVID-19. Several lines of evidence contribute to demonstrating that the etiological diagnosis of SARS-CoV-2 infection (i. e., the coronavirus responsible for COVID-19), will not be possible without laboratory testing, either by directly identifying the presence of the virus in biological samples with (real-time) reverse transcriptase-polymerase chain reaction (RT-PCR), or through detection of immunological response against the virus by monitoring antibody response [3]. Albeit the diagnostic accuracy of RT-PCR on oropharyngeal and nasopharyngeal swabs, and serological testing in serum or plasma used alone is suboptimal, especially within 1 week from symptom onset (i. e., 67% and 38%, respectively), the combination of these two diagnostic techniques may consistently enhance the sensitivity of laboratory diagnosis up to 80–90% [7]. Between 8 to 14 days after the onset of the symptoms, the combined sensitivity of viral RNA identification and antibodies detection increases up to 97%, thus plenty satisfying optimal diagnostic criteria.

Beside the essential, virtually unavoidable, contribution to diagnosing SARS-CoV-2 infection, laboratory medicine is then essential for risk stratification, whereby many tests such as the complete blood cell count, inflammatory, cardiac, muscle, liver, kidney and hemostasis biomarkers, are essential elements for identifying a subset of patients at enhanced risk of developing the most severe COVID-19 complications such as acute respiratory distress syndrome (ARDS), severe inflammatory response syndrome (SIRS), targeted and multiple organ failure (MOF), up to death [8].

At this point in time, it cannot be denied, either by policymakers or hospital administrators, that that COVID-19 outbreak has placed a formidable strain on a health care sector already close to a chasm. Although neither using a crystal ball we will be able to predict if, nor when and how, this unprecedented pandemic will terminate, some lessons must be learned. The local response of laboratory medicine services to the COVID-19 outbreak has been always efficient all around the world, despite the many and reiterated cuts. All professional figures working in clinical laboratories staff shall hence be gloried and prized for providing such an efficient support. Nonetheless, ordinary working with minimally sufficient resources has obligated to urgent recruitment of personnel, often with rapid selections, as well as to the need of purchasing additional instrumentation to meet the increased volume of testing, especially in certain areas of the laboratory such as molecular biology and serology. Shortage of some reagents, due to impossibility to stockpile (for limiting contingent hospital costs) or inability of supply importers to fulfill the demand, has further amplified the vulnerability of this sector. Last but not least, the incessant consolidation of small laboratories into larger facilities has contributed to amplify the diagnostic delay, and hence derange the clinical decision making in many worldwide areas [[9].

Therefore, we conclude here reporting the foremost and celebrated Latin mantra “*herrare humanum est, perseverare autem diabolicum*”, in the most sincere wish that everybody will learn from this exceptional tragedy.
